# Hepatitis C and pulmonary fibrosis

**Published:** 2011-02-01

**Authors:** Rasoul Aliannejad, Mostafa Ghanei

**Affiliations:** 1 Respiratory Diseases and TB Research Center of Guilan University of Medical Science, Razi Hospital, Rasht, IR Iran; 2 Chemical Injuries Research Center, Baqiyatallah University of Medical Sciences, Tehran, IR Iran

**Keywords:** Hepatitis C, Pulmonary fibrosis

## Abstract

**Background:**

Hepatitis C virus (HCV) is a hepatotropic and lymphotropic virus that causes hepatic and extrahepatic disease. Emerging clinical data suggest that chronic HCV infection can lead to many direct and indirect effects on the lung.

**Objectives:**

This article discusses evidence on the relationship between HCV infection and pulmonary fibrosis to increase knowledge on this topic among clinicians and scientists and highlights the need for further study.

**Methods:**

We searched the MEDLINE, ISI WEB OF KNOWLEDGE, OVID, ELSEVIER, and MDCONSULT databases and top respiratory journals, such as the American Journal of Respiratory and Critical Care, Chest, and Thorax for articles in English using the following keywords: hepatitis C, HCV infection, IPF, pulmonary fibrosis, and interstitial pneumonitis. We reviewed the reference lists of all identified studies.

**Results:**

The evidence for a pathogenetic link between pulmonary fibrosis and HCV is: the higher frequency of HCV markers in IPF patients, an increase in lymphocyte and neutrophil numbers in bronchoalveolar lavage of chronic HCV infection patients, and the development of IPF in HCV-related chronic hepatitis that is treated with interferon. There is a discrepancy between studies on the frequency of HCV in IPF patients, which might be attributed to geographical differences of in the prevalence of HCV infection, selection bias in choosing the control group, and the HCV genome.

**Conclusions:**

BAL studies in HCV infection are associated with increased counts of lymphocytes and neutrophils in BAL fluid. These studies show that HCV infection is associated with nonspecific pulmonary inflammatory reactions that are not compatible with IPF but that it can lead to pulmonary fibrosis. The other factor is interferon therapy. Interstitial pneumonia and sarcoidosis are well-documented complications of IFN therapy. More extensive cohort studies should be conducted to confirm an actual causal relationship between HCV infection and pulmonary fibrosis.

Hepatitis C virus (HCV) is a hepatotropic and lymphotropic virus that can cause hepatic and extrahepatic disease. Mixed cryoglobulinemia and non-Hodgkin's lymphoma are the two most common extrahepatic conditions that are linked most closely to HCV. Dermatological, nephrological, neurological, endocrinological, cardiocirculatory, and pulmonary disorders have also been associated with HCV infection [1][2]. Therefore, chronic HCV infection is a systemic disease that has a wide range of clinical manifestations.

Emerging clinical data suggest that chronic HCV infection can lead to many direct and indirect effects on the lung. The direct effects include initiation or exacerbation of preexisting asthma [3][4] and COPD [5][6], interstitial pneumonitis, and pulmonary fibrosis. Indirect effects comprise cirrhosis of the liver (due to HCV) with complications (hepatopulmonary syndrome [7], port pulmonary hypertension [8], mixed cryoglobulinemia [9][10][11], Sicca syndrome [12], non-Hodgkin's B-cell lymphomas [13], autoimmune thyroid disease [14], and myositis [15]).

The pathogenetic link between pulmonary fibrosis and HCV has been evidenced by the higher frequency of HCV markers in IPF patients, an increase in lymphocyte and neutrophil numbers in bronchoalveolar lavage of HCV chronic infection patients, and the development of IPF in HCV-related chronic hepatitis that is treated with interferon. Three years after the discovery of hepatitis C, Japanese investigators measured HCV antibodies in patients with idiopathic pulmonary fibrosis (IPF) and observed a higher prevalence of serum antibodies to HCV in patients with IPF (28.8%) than in age-matched control subjects (3.6%) [16]. Ohta et al. examined the involvement of HCV in the pathogenesis of idiopathic pulmonary fibrosis (IPF). They measured anti-HCV antibodies by ELISA in the sera of 66 IPF patients and observed a high anti-HCV levels in IPF compared with 9464 age-matched controls. Also, 12.2% of ELISA-positive patients were positive by RIBA test, and 14.3% of those who were pathologically diagnosed with UIP were positive for HCV by RT-PCR [17]. Irving et al. examined the involvement of hepatitis C virus (HCV) infection in the pathogenesis of IPF, as suggested by Japanese researchers. They assessed the sera of 62 IPF patients using two second-generation anti-HCV ELISAs and found that only one sample was reactive, which was negative for HCV RNA by RT-PCR.

Consequently, they concluded that HCV infection was no more prevalent in British patients with IPF than in the general population [18]. Subsequently, Meliconi et al. tested the same hypothesis in Italy. Antibodies to HCV were measured in 60 patients with IPF, 130 patients with non-interstitial lung disease, and 4614 blood donors by ELISA and RIBA, and HCV-RNA was assessed by PCR. The prevalence of HCV infection and viral replication increased in Italian IPF patients, but the levels of anti-HCV antibodies did not differ versus other lung diseases [19]. Similarly, Ferri et al. monitored the presence of lung disease in 300 patients with chronic HCV infection and found that 8 patients showed signs of interstitial lung involvement by high-resolution CT (HRCT). The patients had varying degrees of DLCO reduction, and the percentage of neutrophils in BALF increased in 4 patients. One patient died from a rapidly progressive respiratory disorder, 2 patients deteriorated gradually, and 5 patients remained stable [12]. A recent retrospective cohort study was performed in 6150 Japanese HCV-infected patients and 2050 hepatitis B virus patients (as a control group). The mean observation period was 8.0 ± 5.9 years in the HCV group and 6.3 ± 5.5 years in the HBV group. The cumulative rates of IPF development in the HCV group were 0.3% at Year 10 and 0.9% at Year 20. The prevalence of IPF was slightly higher in the HCV group compared with the HBV group, and the rate of IPF in HCV patients was significantly greater in patients aged ≥ 55 years, patients who had a smoking index ≥ 20, and patients with cirrhosis [20].

The discrepancy between these results has several explanations. The first is geographical differences in the prevalence of HCV infection, which is high in Japan and Mediterranean countries and low in northern Europe. Also, people who volunteer as blood donors are generally not at risk for hepatitis viruses; thus, they do not represent the most appropriate control group since they were younger. Further, the HCV genome should be considered with regard to this discrepancy. Keishi Kubo et al. studied BALF cell counts in 13 Japanese patients with active chronic hepatitis C and 13 healthy volunteers. There was no difference in total cell counts in lavage fluid between the groups, but lavage lymphocyte and eosinophil numbers were higher in patients with chronic hepatitis C [21].

Yamaguchi et al. performed a prospective nonrandomized study on BAL fluid in Japanese patients with chronic hepatitis C before and after treatment with interferon alpha and healthy controls. Lymphocyte counts in the BAL fluid increased significantly in both groups before and after treatment compared with controls. Activated T cell (HLA-DR-positive) counts rose in the pretreatment group versus the controls but fell after treatment compared with the pretreatment count. These findings suggest that HCV infection is associated with increased counts of lymphocytes and neutrophils in BAL fluid and that treatment with IFN-alpha appears to alter lymphocyte surface markers [22].

Idilman et al. conducted the same study in 18 Turkish patients with chronic hepatitis C and 14 healthy volunteers. One patient (5.6%) only had HCV RNA in the bronchoalveolar lavage. The total cell and neutrophil counts in the BAL fluid were significantly higher in patients with chronic hepatitis C compared with controls. No difference was observed in the percentage of lymphocytes, macrophages, eosinophils, or T cell subsets or B cell numbers in BAL fluid between the groups [23]. These studies indicated disparities in the cellular content of BAL fluid in HCV hepatitis, some of which generated different results in comparison with BAL fluid features of IPF. BAL fluid of IPF patients contain an excess of neutrophils, and lymphocytosis and eosinophil content of more than 20% in BAL fluid are not hallmarks of UIP/IPF [24]. These studies on BALF cellular content were performed in HCV patients without evidence of pulmonary involvement. These reports demonstrate that HCV infection can be associated with nonspecific pulmonary inflammatory reactions that are not compatible with IPF but that it can lead to pulmonary fibrosis.Additional evidence of interstitial involvement in chronic HCV infection has come from measurements of epithelial permeability by TC 99m -labeled diethylene tri amin pentaacetic acid aerosol scintigraphy. HCV-positive patients without clinical pulmonary symptoms have significantly increased epithelial permeability compared with controls, suggesting early interstitial lung disease [25].

Interferon therapy should also be considered with regard to the link between pulmonary fibrosis and HCV. IFN has been used successfully to treat chronic HCV infection, but it can cause pulmonary complications. Interstitial pneumonia and sarcoidosis are well-documented complications of IFN therapy [26][27][28]. The exact mechanism of HCV in the pathogenesis of interstitial pneumonitis is not understood, but chronic immune activation and inflammation that is induced by HCV infection may be involved. Because HCV induces chronic inflammation and fibrosis in the liver, HCV is believed to have similar functions in the lung and might mediate the pathogenesis of pulmonary fibrosis. The other possible mechanism is that antigens and antibodies from the bowel or other organs enter portal circulation and are not separated sufficiently in patients with severe liver dysfunction. Immune complexes that are formed by these antigens and antibodies enter systemic circulation and accumulate in the glomeruli or lung [29]. Arase et al. examined the lung and kidney by immunofluorescence microscopy; they did not detect immunoglobulin in formalin-fixed lung tissue but detected it in the glomeruli of patients with mesangial and membranoproliferative glomerulonephritis [29]. These results indicate that serum immunoglobulin has a minor role in IPF. However, there might have been low sensitivity due to the use of formalin-fixed tissue. The function of in?ammation in the development of IPF remains unresolved. Histologically and radiologically (ground glass densities of IPF by CT), there is modest evidence to support the involvement of inflammation in IPF patients. IPF may occur in susceptible individuals with abnormal lung repair mechanisms. Therefore, several environmental insults, such as viral infections, could effect and perpetuate IPF. However, in?ammation can lead to ?brosis in other clinical entities [30]. Conversely, some conditions, such as MC and sicca syndrome, are observed in HCV infection and can involve the lung with or without clinical symptoms [9][10][12]. Some MC patients may present with dyspnea of exertion, dry cough, interstitial lung ?brosis, pleural effusions, or hemoptysis, which can be a consequence of alveolar hemorrhage. Koike et al. reported that transgenic mice that carry the HCV envelope gene develop exocrinopathy that resembles Sjogren syndrome [31].The incidence of IPF has not been examined in Iran. A recent systematic review has indicated that the prevalence of HCV infection in Iran is 0.16%, which is low compared with other countries [32]. Therefore, this evidence should prompt studies on the correlation or a causal relationship between HCV and IPF. More extensive cohort studies should be conducted to determine whether there is a causal relationship between HCV infection and pulmonary fibrosis.
